# What do photosynthetic organisms need to thrive in all circumstances?

**DOI:** 10.1093/plcell/koad213

**Published:** 2023-08-03

**Authors:** Solène L Y Moulin

**Affiliations:** Assistant Features Editor, The Plant Cell, American Society of Plant Biologists; Department of Pathology, Stanford University School of Medicine, Stanford, CA 94305, USA

Photosynthetic organisms are key players in the global carbon cycle since they convert CO_2_ into organic carbon. Cyanobacteria are great models to study photosynthesis in plants and algae, whose chloroplasts share ancestry. In addition, cyanobacteria are a promising chassis for bio-production. Despite their importance, the most studied cyanobacterium, *Synechocystis* sp. PCC 6803, has only one-half of its genes functionally annotated. Recent advances in new sequencing technologies have opened the door to high-throughput functional genomics. The feasibility of such a technique in cyanobacteria has been proven using a CRISPRi library in *Synechocystis*, but its low guide coverage has led to ambiguous results (Yao et al. 2020). **Rui Miao, Michael Jahn, and colleagues (Miao et al. 2023)** capitalized on this proof of concept to generate an expanded library and tested it across 11 growth conditions compromising variation in carbon and nitrogen source as well as light availability.

The CRISPRi library design comprised multiple single-guide RNAs (sgRNAs) targeting specific genes and noncoding RNAs that are potential regulatory molecules. The pooled library was cultured with high or low CO_2_ and exposed to high-, intermediate-, or low-light intensities. In addition, mixotrophic and photoheterotrophic conditions were tested. Fitness scores were assigned based on changes in sgRNA abundance determined by sequencing throughout a period of cultivation for each condition (Figure). The absolute fitness score of sgRNAs appeared to decline with distance from the promoter; therefore, the weighted mean of sgRNA fitness scores was used as the gene fitness score. The results of this large library competition experiment are available in the interactive web application Shinylib (https://m-jahn.shinyapps.io/ShinyLib/).

**Figure.**
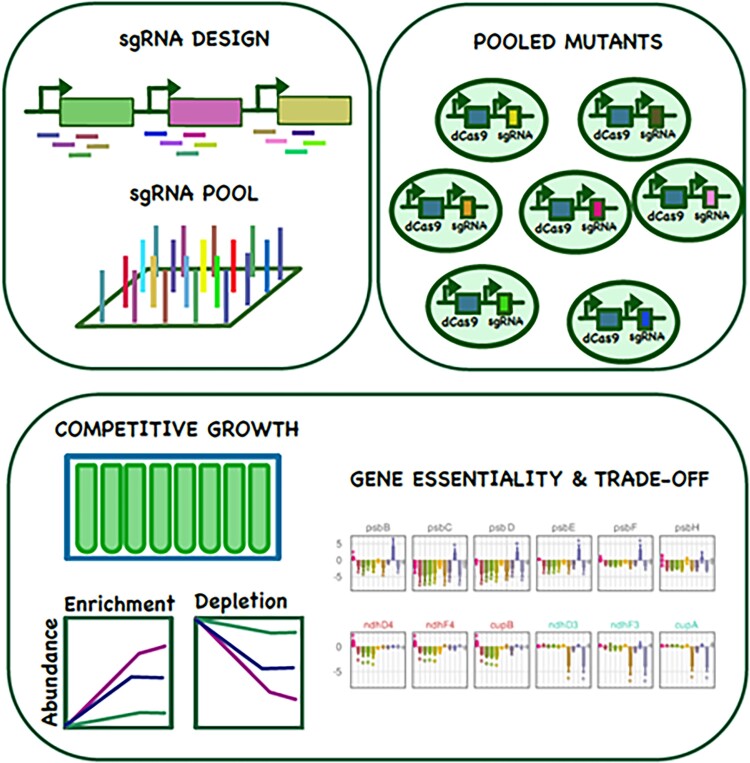
CRISPR interference screens reveal gene essentiality and trade-off. A pooled library was designed with multiple RNA guides targeting every gene in the genome of *Synechocystis* sp. PCC6803. The pooled culture of mutants was tested across 11 growth conditions to extract a fitness score of each individual mutant. Figure credit: Rui Miao.

Most metabolic pathways showed a negative median fitness score across various conditions. The strongest detrimental effect on fitness was associated with repression of protein synthesis pathways and energy metabolism, and carbon metabolic pathway repression exhibited weaker effects. Higher abundance of enzymes, higher proportion of isoenzymes, or compensation by alternative metabolic fluxes might be responsible for the milder phenotypes observed when carbon metabolic pathways are affected. In conditions of extreme light stress that correlate with low growth rate, the fitness penalty for repression of protein synthesis pathways was weaker than in other conditions. As previously described in *Escherichia coli* and cyanobacteria, significant ribosome reserve might be sufficient to sustain protein synthesis (Wu et al. 2022), leading to insensitivity to the CRISPRi.

Analysis of genes involved in photosynthesis revealed fitness tradeoffs for anticipating light stress and carbon limitation. In conditions where a protein is expected not to be needed, repression of its expression showed a beneficial fitness effect. These results suggest a level of preparedness for a potential shift in light or CO_2_ availability that comes at the expense of growth rate when light and CO_2_ are not limiting.

Metabolic fluxes in the central carbon metabolism have been difficult to determine due to the presence of gene duplications or isoenzymes that can theoretically compensate for one another. Comparison between photoautotrophy, mixotrophy, and photoheterotrophy can show compensation points and essential reactions because the repression of the latter will have a more dramatic effect. Repression of the oxidative pentose phosphate pathway appeared to have a high fitness penalty specifically in photoheterotrophic growth. On the contrary, repression of the TCA cycle had nearly no effect on fitness in all conditions. However, these results must be interpreted with caution because high enzyme abundance could sustain a “reserve flux capacity” upon repression of the gene expression as illustrated by Rubisco repression.

Out of all the ncRNA tested, only a few showed a fitness effect upon repression. Fitness score of antisense RNAs and internal transcription start sites appeared to be correlated with the fitness of the associated gene. Many sRNA targets were important for growth, but all their fitness scores correlated with the fitness score of the surrounding genes in the genome with the exception of only 2 ncRNAs: Ncr1080 and Ncr0050. Ncr1080 has previously been identified as an independent transcriptional unit upregulated in high light (Kopf et al. 2014), but Ncr0050 has not been characterized.

Overall, this study provides a large data set to explore the physiology of cyanobacteria and more broadly of photosynthetic organisms that will drive future research. It reveals suboptimal regulation and essential genes depending on conditions that are of interest for use of cyanobacteria for sustainable production of valuable chemicals. In addition, the large data set allowed a retrospective analysis identifying “design rules” for effective guide RNAs in *Synechocystis*, which is a great resource for the community.

## Dive Curated Terms

The following phenotypic, genotypic, and functional terms are of significance to the work described in this paper:
